# *Anaerobutyricum* and *Subdoligranulum* Are Differentially Enriched in Broilers with Disparate Weight Gains

**DOI:** 10.3390/ani13111834

**Published:** 2023-06-01

**Authors:** Jing Liu, Kelsy Robinson, Wentao Lyu, Qing Yang, Jing Wang, Karen D. Christensen, Guolong Zhang

**Affiliations:** 1 Department of Animal and Food Sciences, Oklahoma State University, Stillwater, OK 74078, USA; jing.liu12@okstate.edu (J.L.); kelsy.robinson@usda.gov (K.R.); lvwt@zaas.ac.cn (W.L.); qing.yang@okstate.edu (Q.Y.); wangjing976119@163.com (J.W.); 2 Poultry Research Unit, USDA–Agricultural Research Service, Starkville, MS 39759, USA; 3 State Key Laboratory for Managing Biotic and Chemical Threats to the Quality and Safety of Agro-Products, Institute of Agro-Product Safety and Nutrition, Zhejiang Academy of Agricultural Sciences, Hangzhou 310021, China; 4 College of Animal Science and Technology, China Agricultural University, Beijing 100193, China; 5 Institute of Animal Husbandry and Veterinary Medicine, Beijing Academy of Agriculture and Forestry Sciences, Beijing 100097, China; 6 Tyson Foods, Springdale, AR 72762, USA; karen.christensen@tyson.com

**Keywords:** body weight, microbiota, microbiome, 16S rRNA gene sequencing, chickens

## Abstract

**Simple Summary:**

The intestinal microbiota plays a vital role in nutrient digestion, pathogen exclusion, immune de-velopment, and subsequently animal productivity. However, specific microbes that are associated with animal growth are still unclear. Here we profiled the cecal microbiota to identify those bacteria that are linked to body weight gain of broiler chickens raised under typical commercial conditions. Several bacteria were found to show either a positive or negative association with body weight. These bacteria may be further explored to improve the growth performance of chickens and also as potential biomarkers for the selection of broiler chickens with different growth rates.

**Abstract:**

The intestinal microbiota is critically important for animal health and productivity. However, the influence of the intestinal microbiota on animal growth efficiency remains elusive. This current study was aimed at identifying the intestinal bacteria that are associated with the growth rate of broilers in a commercial production setting. Ross 708 broilers with extremely high, medium, and extremely low body weight (BW) were separately selected for each sex from a house of approximately 18,000 chickens on day 42. The cecal content of each animal was subjected to 16S rRNA gene sequencing for microbiota profiling. Our results indicate that a number of bacteria were differentially enriched among different groups of broilers, with several showing a significant correlation (*p* < 0.05) with BW in both sexes or in a sex-specific manner. *Subdoligranulum* was drastically diminished in high-BW birds with a strong negative correlation with BW in both males and females. While one *Anaerobutyricum* strain showed a positive correlation with BW in both sexes, another strain of *Anaerobutyricum* was positively correlated with BW only in females. These sex-dependent and -independent bacteria could be targeted for improving the growth efficiency and may also be explored as potential biomarkers for the growth rate of broiler chickens.

## 1. Introduction

Chicken is an important source of animal protein consumed worldwide, and the demand for chicken products has grown rapidly in recent decades [1]. To meet increasing global demands, maximizing growth performance while preserving animal health and welfare standards is vitally important for the poultry industry. The intestinal microbiota is critical to nutrient utilization, energy retention, host immune response, and health of chickens [2,3]. To enhance growth performance of chickens, it is desirable to identify and modulate those bacteria that are involved in nutrient utilization, energy retention, and ultimately animal growth efficiency [2]. 

Several studies have investigated the relationship between the intestinal microbiota and body weight (BW) of chickens; however, the findings have been inconsistent thus far and different bacteria have been reported in different studies [4,5,6,7,8,9]. The reasons could be due to the variations in the environment (e.g., feed, housing, litter, and management), host (e.g., breeds, sex, and age), and sample size among different studies because each of these factors has a profound impact on the composition and function of the intestinal microbiome [10]. At the same time, the applicability of these results to commercial poultry production practices may be questionable because the majority of the studies are conducted on small research farms, where the environment is markedly different from commercial production conditions. As such, it is crucial to examine the relationship between intestinal microbiota and BW in flocks that are raised under a typical commercial setting. 

Furthermore, most of the studies classified bacteria to the level of genus or operational taxonomic units (OTUs), which are represented by a group of bacteria whose 16S rRNA gene shows a sequence identity of 97% and above [11]. Newly-developed bioinformatic tools, such as Deblur [12], now allow the separation of amplicon sequence variants (ASVs) at single-nucleotide resolution [13]. The goal of this study was, therefore, to employ the up-to-date reference rRNA database and bioinformatic tools to investigate the relationship between the intestinal microbiota and BW in a commercial flock of straight run broilers containing both sexes.

## 2. Materials and Methods

### 2.1. Animals and Sample Collection

A flock of approximately 18,000 male and female Ross-708 broilers were raised in a single house in the Applied Broiler Research Farm at the University of Arkansas (Fayetteville, AR) under industrial standard management. A crumbled commercial starter diet (Simmons Food, Siloam Springs, AR, USA) was provided from day 0–12 and switched to a pelleted grower diet from day 13–28, and a finisher diet from day 29 till the market age, day 42. Animals had ad libitum access to feed and water throughout the trial. On day 42, 600 apparently healthy broilers were randomly chosen and weighed to represent the entire house. To select the animals with the largest BW difference, 10 broilers of each sex with the highest, medium, and lowest BW were chosen. All 60 selected broilers were then euthanized via cervical dislocation. Approximately 0.2–0.5 g of the cecal content was aseptically collected and snap frozen in liquid nitrogen and stored at −80 °C until DNA extraction.

### 2.2. DNA Extraction and 16S rRNA Gene Sequencing

Microbial DNA in the cecal contents was extracted using Quick-DNA Fecal/Soil Microbe Miniprep Kit (Zymo Research, Irvine, CA, USA) according to the manufacturer’s instructions. DNA concentration and quality were measured using NanoDrop ND-1000 (Wilmington, DE, USA), followed by commercial 16S rRNA gene sequencing by Novogene (Beijing, China). Briefly, the V3-V4 region of the bacterial 16S rNRA gene was amplified using primers (341F: CCTAYGGGRBGCASCAG and 806R: GGACTACNNGGGTATCTAAT). A library was prepared using NEBNext^®^ Ultra™ Library Prep Kit (New England Biolabs, Ipswich, MA, USA) and subjected to PE250 sequencing on an Illumina HiSeq platform.

### 2.3. Bioinformatics and Statistical Analysis

Raw DNA sequencing reads were analyzed using the QIIME 2 pipeline (v. 2020.11; https://qiime2.org/ (accessed on 11 February 2023)). Briefly, adaptor and primer sequences were removed from each read using the cut-adapt plugin. Paired-end reads were then merged using ‘vsearch join-pairs’ and low-quality reads were filtered out using ‘quality-filter q-score’. Sequences were trimmed to 403 nucleotides and denoised using Deblur [12]. The resulting sequences were then classified into bacterial ASVs using the RDP 16S rRNA training set (v. 18) and Bayesian classifier. A bootstrap confidence of 80% was used for taxonomic classification. ASVs with a classification of <80% were assigned the name of the last confidently assigned level followed by “_unidentified”. ASVs appearing in <5% of samples were removed from analysis. Top 20 ASVs and all differentially enriched bacteria were further confirmed and reclassified, if necessary, based on a more recent EzBioCloud 16S database (v. 2021.07.07). Data were normalized using cumulative sum scaling (CSS) in the metagenomeSeq package of R (v. 1.4.0) [14].

The α-diversity (Shannon’s Index, Observed ASVs, and Pielou’s Evenness) and β-diversity (unweighted and weighted UniFrac distances) were calculated using the phyloseq package in R (v. 1.42.0) [15]. Statistical significance in α-diversity and relative abundance were determined using non-parametric Kruskal-Wallis test. Significance in β-diversity was determined using non-parametric permutational multivariate analysis of variance (PERMANOVA) using the adonis function in the vegan package (v. 2.6.4) [16]. Differential enrichment of bacteria between high, medium, and low groups for males and females were obtained using linear discriminant analysis (LDA) effect size (LEfSe) with *p* < 0.05 and an LDA score of ≥3.0 as the threshold [17]. Spearman correlation analysis was further performed to identify the correlation between differentially enriched ASVs and BW of broilers using the psych package (v. 1.9.12.31). Associations were considered significant if *p* < 0.05 and |R| ≥ 0.3.

## 3. Results

### 3.1. Body Weight and Cecal Microbial Diversity and Composition of Broilers

**Figure 1:**
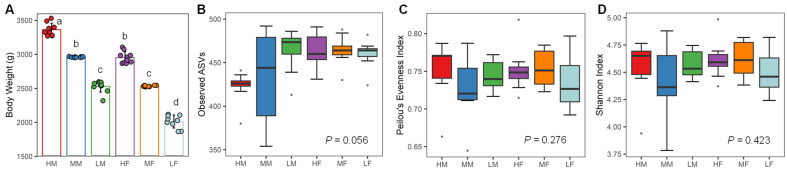
Body weight (BW) and α-diversity of the cecal microbiota among different groups of Ross-708 broiler chickens. Ten broilers of each sex with the highest, medium, and lowest BW were selected on day 42. The cecal contents were subjected to 16S rRNA gene sequencing. (**A**) BW of different groups of chickens. Bars with different superscripts are statistically significant (*p* < 0.01 by one-way ANOVA and post hoc Tukey’s test). Observed ASVs (**B**); Pielou’s Evenness (**C**); and Shannon Index (**D**) were calculated to measure α-diversity of the cecal microbiota and Kruskal-Wallis test was used for statistical significance determination. HM = high male, MM = medium male, LM = low male, HF = high female, MF = medium female, and LF = low female.

Among a total of 60 day-42 male and female Ross-708 chickens selected from a house of approximately 18,000 broilers, high-quality sequencing results were obtained with 58 of the cecal content samples. BW of these 58 chickens were drastically different among different groups (*p* < 0.01), averaging 3365.6 ± 85.2, 2960.7± 5.4, 2529.2 ± 79.2, 2953.1 ± 81.0, 2536.8 ± 8.8, 2017.9 ± 86.4 among high males (HM), medium males (MM), low males (LM), high females (HF), medium females (MF), and low females (LF), respectively (Figure 1A).

After removing low-quality reads and chimeras using QIIME 2, a total of 986,002 reads were retained for 58 cecal DNA samples, with an average of 17,000 ± 5032 sequences per sample. The sequences were assigned to 1904 ASVs, and 586 ASVs were retained after removing those present in <5% of samples. Comparisons of Observed ASVs (Figure 1B), Pielou’s Evenness (Figure 1C), and Shannon Index (Figure 1D) among different BW groups of male and female chickens revealed no significant differences (*p* > 0.05).

**Figure 2:**
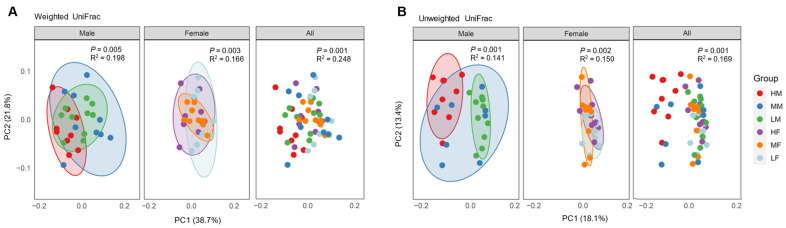
The β-diversity of the cecal microbiota among different groups of day-42 Ross-708 broiler chickens. Weighted UniFrac (**A**) and unweighted UniFac distances (**B**) were used to generate principal coordinates analysis (PCoA) plots. Permutational multivariate analysis of variance (PERMANOVA) was used for statistical significance determination. HM = high male, MM = medium male, LM = low male, HF = high female, MF = medium female, and LF = low female.

To further compare the differences in the microbiota among different groups, principal coordinates analysis (PCoA) was performed based on weighted UniFrac (Figure 2A) and unweighted UniFrac distances (Figure 2B). In male chickens, PERMANOVA analysis revealed significant separation among different groups in both weighted UniFrac (*p* = 0.005, R^2^ = 0.198) and unweighted UniFrac distances (*p* = 0.001, R^2^ = 0.141). Significant differences were also observed among different groups in both weighted (*p* = 0.003, R^2^ = 0.166) and unweighted UniFrac indices (*p* = 0.002, R^2^ = 0.150) for females. Pairwise tests further revealed significance (*p* < 0.05) in both male and female chickens between high and low-BW groups for both weighted and unweighted UniFrac distances (Table 1). Because of this observation supported by published evidence showing the influence of intestinal microbiota by sex [6,18], males and females were not combined for each BW group for subsequent analyses.

**Figure 3:**
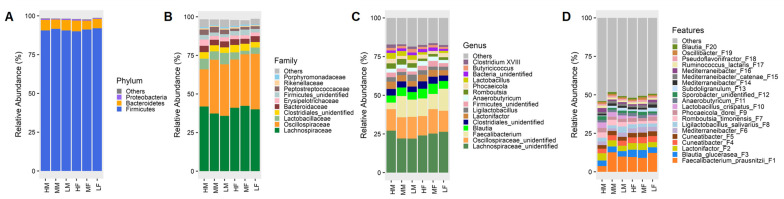
The composition of the cecal microbiota among different groups of day-42 Ross-708 broiler chickens. Average relative abundances (%) of top 3 phyla (**A**); top 10 families (**B**); top 15 genera (**C**); and top 20 ASVs (**D**) are shown. HM = high male, MM = medium male, LM = low male, HF = high female, MF = medium female, and LF = low female.

Compositionally, a total of 6 phyla, 14 classes, 20 orders, 32 families, and 79 genera were identified in 58 cecal content samples using the RDP database. Firmicutes and Bacteroidetes were the two most dominant phyla in both male and female chickens, representing 96–98% in each group (Figure 3A). At the family level, *Lachnospiraceae* was the most abundant at 36–42%, followed by *Oscillospiraceae* (24–36%) in both male and female chickens (Figure 3B). At the genus level, an unclassified genus in each of the *Lachnospiraceae* and *Oscillospiraceae* families accounted for 22–27% and 12–16%, respectively, followed by *Faecalibacterium* (4–14%) and *Blautia* (5–7%) (Figure 3C). The cecal microbiota was highly diverse, with top 20 ASVs accounting for approximately 50% of all bacteria, while the most abundant bacterium was *Faecalibacterium praunsnitzii* F1 (4–13%) (Figure 3D).

### 3.2. Differential Enrichment of Bacteria among Different Groups

**Figure 4:**
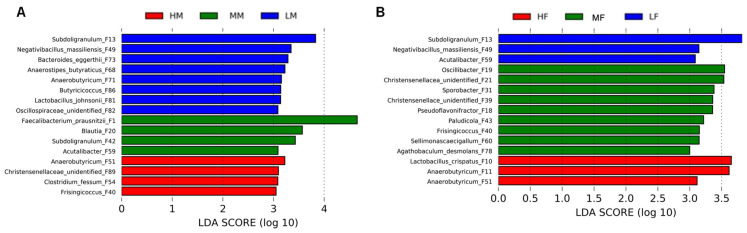
Differential enrichment of the top 100 cecal bacterial ASVs among different groups of male (**A**) and female chickens (**B**). LEfSe analysis was performed using *p* < 0.05 and a LDA score of ≥3.0 as the threshold. HM = high male, MM = medium male, LM = low male, HF = high female, MF = medium female, LF = low female, and LDA = linear discriminant analysis.

LEfSe analysis [17] was used to identify specific bacterial ASVs that were enriched in different BW groups of male and female broilers. Using a threshold LDA score of 3.0, a total of 16 ASVs were identified to be differentially enriched among three BW groups of male broilers (Figure 4A). Among these, *Anaerobutyricum* F51, *Clostridium fessum* F54, *Frisingicoccus* F40, and an unidentified *Christensenellaceae* member F89 were significantly enriched in the HM group, while *F. praunsnitzii* F1, *Blautia* F20, *Subdoligranulum* F42, and *Acutalibacter* F59 were more abundant in the MM group. Furthermore, four members of *Oscillospiraceae* (*Subdoligranulum* F13, *Negativibacillus massiliensis* F49, *Butyricicoccus* F86, and an unclassified *Oscillospiraceae* member F82), as well as two members of *Lachnospiraceae* (*Anaerostipes butyraticus* F68 and *Anaerobutyricum* F71), were enriched in the LM group (Figure 4A).

Among female broilers, a total of 15 ASVs were differentially enriched among different BW groups (Figure 4B). Specifically, two members of *Anaerobutyricum* (F11 and F51), as well as *Lactobacillus crispatus* (F10), were enriched in the HF group, while *Subdoligranulum* F13, *N. massiliensis* F49, and *Acutalibacter* F59 were enriched in the LF group. Another nine ASVs were also found to be more abundant in the MF group. Notably, *Subdoligranulum* F13 and *N. massiliensis* F49 were commonly enriched in low-BW groups in both sexes, while *Anaerobutyricum* F51 was enriched in high-BW groups both male and female chickens (Figure 4A,B).

### 3.3. Correlations between Intestinal Microbiota and Body Weight

**Figure 5:**
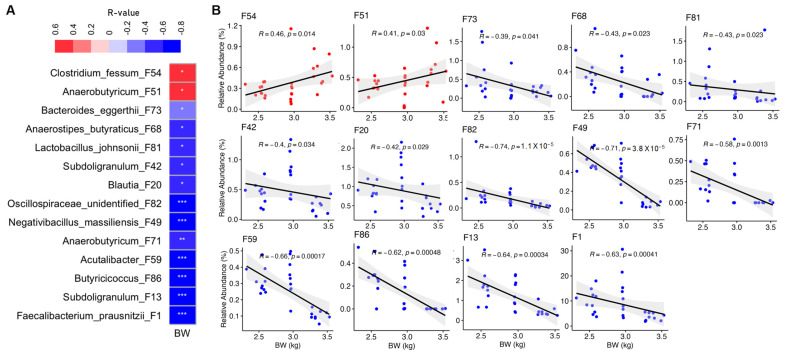
Spearman correlation between BW and relative abundance (%) of bacterial ASVs in male chickens. (**A**) Correlation coefficient and significance between BW and differentially enriched ASVs identified from LEfSe analysis. Only those features with *p* < 0.05 and |R| > 0.30 are shown. * *p* < 0.05, ** *p* < 0.01, *** *p* < 0.001. (**B**) Scatterplots of individual bacterial ASVs showing a significant correlation with BW. Only those with *p* < 0.001 are displayed for ASVs showing a negative correlation. The solid line in each graph represents the line of best fit, while gray shading indicates the 95% confidence interval. In a few cases, 1–3 outliers were omitted for the sake of better visualization.

**Figure 6:**
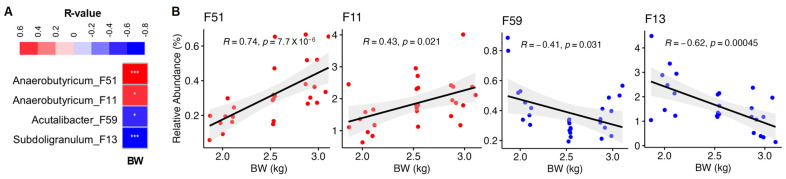
Spearman correlation between BW and relative abundance (%) of bacterial ASVs in female chickens. (**A**) Correlation coefficient and significance between BW and differentially enriched ASVs identified from LEfSe analysis. Only those features with *p* < 0.05 and |R| > 0.30 are shown. * *p* < 0.05, *** *p* < 0.001. (**B**) Scatterplots of individual bacterial ASVs showing a significant correlation with BW. the solid line in each graph represents the line of best fit, while gray shading indicates the 95% confidence interval.

To further identify bacterial ASVs that are correlated with BW of chickens, we performed Spearman correlation analysis with all differentially enriched ASVs. In male chickens, Spearman correlation confirmed 14 out of 16 ASVs showing a significant positive or negative correlation with BW (Figure 5A), with |R| values ranging from 0.39 to 0.71. Among these, *Anaerobutyricum* F51 and *C. fessum* F54 were positively correlated with BW (*p* < 0.05), while the remaining 12 ASVs showed a significant negative correlation with BW (*p* < 0.05) (Figure 5B). In female chickens, 4 out of 15 ASVs showed a significant positive or negative correlation with BW, with |R| values ranging from 0.40 to 0.74 (Figure 6A). Specifically, two members of *Anaerobutyricum* (F11 and F51) were significantly positively correlated with BW, while *Subdoligranulum* F13 and *Acutalibacter* F59 were negatively correlated with BW (Figure 6B).

**Figure 7:**
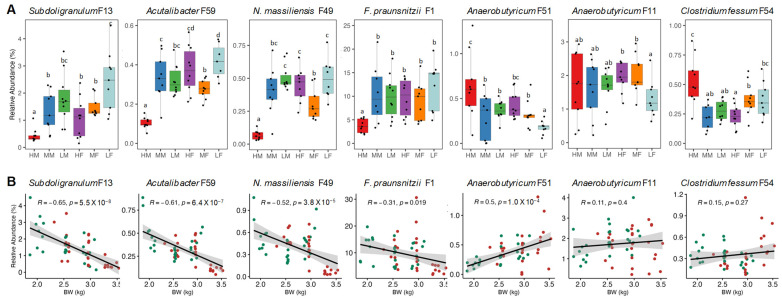
Association of selected bacteria with body weight (BW) of both male and female broilers. (**A**) Relative abundance (%) of selected bacteria in different groups of chickens. HM = high male, MM = medium male, LM = low male, HF = high female, MF = medium female, and LF = low female. Different letters on the bars denote statistical significance (*p* < 0.05) based on Kruskal-Wallis test followed by pairwise Wilcox comparison. (**B**) Spearman correlation between selected bacteria and BW in both sexes of chickens. Solid line in each graph represents the line of best fit, while gray shading indicates the 95% confidence interval. Green dots represent male chickens, while red dots represent female chickens.

Among them, *Subdoligranulum* F13 and *Acutalibacter* F59 largely showed a BW-dependent increase in relative abundance in both males and females (Figure 7A) with a negative correlation with BW, regardless of sex (R > 0.6, *p* < 0.0001) (Figure 7B). A similar trend also occurred with *N. massiliensis* F49, which was enriched in low-BW birds showing a strong negative correlation with BW in both sexes (R = 0.52, *p* < 0.0001) (Figure 7). It is noted that *F. praunsnitzii* F1, closely-related to *Subdoligranulum* [19], was also decreased in high-BW males and tended to decrease in females as well, showing a significant negative correlation with BW if both sexes were combined (R = 0.31, *p* = 0.02) (Figure 7). In contrast, *Anaerobutyricum* F51 was enriched in high-BW chickens in both sexes with a strong positive correlation with BW (R = 0.5, *p* = 0.0001) (Figure 7).

Similar to *Anaerobutyricum* F51, a different *Anaerobutyricum* strain F11 was significantly diminished in low-BW female chickens, but no obvious difference was observed among males of different BW (Figure 7A), and unsurprisingly, no significant overall correlation between *Anaerobutyricum* F11 and BW (Figure 7B), suggesting that a negative correlation between *Anaerobutyricum* F11 and BW may be only limited to females (Figure 6). Conversely, *Clostridium fessum* F54 was enriched in high-BW birds (Figure 4) with a significant positive correlation with BW only among males (Figure 5). However, no such trends with *C. fessum* F54 occurred in females (Figure 7). Overall, these results suggest the existence of both sex-dependent and -independent bacteria that are linked to weight gain in broilers.

## 4. Discussion

To increase chicken production profitability while minimizing its environmental impact, it is crucial to understand the relationship between the intestinal microbiota and animal growth efficiency. While much research has been conducted to explore the association between chicken BW and the intestinal microbiota composition, the outcomes remain highly varied among different studies [4,5,6,7,8,9]. For example, Rubio et al. [4] observed a trend of positive correlation between lactobacilli in the cecum and body weight (BW) in male broilers, while Han et al. [5] found cecal *Lactococcus* is positively correlated with BW, but *Anaerovibrio*, *Prevotella*, and *Akkermansia* are negatively correlated with BW. On the other hand, Lee et al. [6] reported an enrichment of *Faecalibacterium* and *Shuttleworthia* in high-BW chickens, while Zhou et al. [8] found that *Alistipes putredinis*, *Faecalibacterium praunsnitzii, Lactobacillus crispatus*, *L. ingluviei*, *L. salivarius, Subdoligranum variabile,* and unclassified species of *Parabacteroides, Collinsella*, and *Olsenella* are enriched in the cecum of high-BW chickens. On the other hand, Farkas et al. [9] revealed a negative correlation between BW and several bacteria such as *Negativibacillus, Defluviitaleaceae* UCG-011, *Butyricicoccus*, *Ruminiclostridium-9, Ruminococcaceae* UCG-013, GCA-900066575, and *Bilophila* in the cecum of high-BW chickens, while no bacteria were found to be positively correlated with BW. Such large variations among different studies are likely due to the small-scale nature and a lack of selection strength for BW in most studies.

Additionally, only male broilers were used in the studies [4,5,9], while males and females were combined in the microbiome analysis in the study [8]. Furthermore, the ages of animals where intestinal contents were collected varied from days 17, 21, 35, and 37 to day 245 [4,5,6,7,8,9]. Because of these huge differences, it is no surprise that there are still no definitive conclusions on growth-associated microbes. In this study, we attempted to select animals with highly disparate growth trajectories from a commercial house of approximately 18,000 broilers and further separate them by sex for subsequent deep sequencing and bacterial classification at single-nucleotide resolution.

Although we observed no obvious differences in α-diversity of the cecal microbiota among different BW groups, β-diversity varied significantly among different groups and between males and females, indicating the influence of both BW and sex on the microbial community composition. No differences in bacterial richness among high, medium, and low-BW chickens were reported earlier [6]; however, other studies reported an increased α-diversity in high-BW chickens [8]. A clear difference in β-diversity between low and high-BW chickens was also observed [8,9].

Using LEfSe and Spearman correlation analysis, we have identified a number of differentially enriched bacterial ASVs among different groups and found that many are correlated with BW in both sex-dependent and sex-independent manners. Among those differentially enriched ASVs, we have found in this study that at least four BW-linked bacteria are shared between male and female chickens. *Subdoligranulum* F13, *N. massiliensis* F49, *and Acutalibacter* F59 show a strong negative correlation with BW, while *Anaerobutyricum* F51 is positively correlated with BW in both sexes. Interestingly, another *Anaerobutyricum* strain F11 appears to be in a positive correlation with BW only in females. On the other hand, *Clostridium fessum* F54 is enriched in high-BW group and positively correlated with BW only in males.

*Subdoligranulum* is a strictly anaerobic, Gram-negative bacterium in the *Oscillospiraceae* family. *S. variabile*, the only species of this genus isolated and described so far, has been shown to produce butyrate [20]. In our study, *Subdoligranulum* is the most differentially abundant in the cecum of low-BW groups of both males and females, with relative abundances being 1.80% and 0.44% on average in low and high-BW male broilers, respectively. In female birds, *Subdoligranulum* F13 accounted for 2.45% and 1.06% in low and high-BW groups, respectively. A negative association between *Subdoligranulum* and BW is consistent with an earlier finding that *S. variabile* is negatively correlated with fat mass and adipocyte diameter in humans [21]. We also revealed earlier that relative abundance of *Subdoligranulum* in the ileum was negatively associated with feed efficiency in broilers [22].

Interestingly, a strain of *Gemmiger,* a genus closely related to *Subdoligranulum* known as the *Gemmiger/Subdoligranulum* cluster [23], was also reported to show the highest differential enrichment by approximately 36-fold in low-BW male broilers (6.19%) compared to high-BW ones (0.17%) [7]. However, the same study also showed four other much less abundant *Gemmiger* strains to be slightly enriched in high-BW male birds, although no female broilers were investigated in the study [7]. Similar to *Subdoligranulum* [21], *Gemminger* is significantly decreased in overweight and obese humans [24,25], which is consistent with the diminishment of *Subdoligranulum* in high-BW broilers and its negative correlation with BW that we observed in this study. These results reinforce the notion that *Gemmiger* and *Subdoligranulum* may affect host by interfering with lipid metabolism and fat deposition. However, additional research is warranted to better understand the involvement of *Gemmiger/Subdoligranulum* in regulating host metabolism and BW.

Perhaps to further strengthen our conclusion on the negative association between *Gemmiger/Subdoligranulum* and BW, another closely related bacterium, *F. prausnitzii* [19], the most dominant bacterium in the cecum in our study, is also more abundantly present in low-BW chickens, particularly among males. *F. prausnitzii* is well known to be anti-inflammatory and reduced in overweight and obese human patients [26,27]. However, these observations are in direct contradiction to two earlier studies [6,8], which showed *Faecalibacterium* and *Subdoligranulum* to be enriched in high-BW chickens. Such a discrepancy is currently unknown.

*N. massiliensis* is a member of the *Oscillospiraceae* family and is another bacterium that shows a negative correlation with the BW of broilers of both sexes, which is consistent with an earlier report on a negative correlation between cecal *Negativibacillus* and BW in male broilers [9]. Larzábal et al. [28] also found that the relative abundance of *Negativibacillus* on the rectum mucosa was increased in *E. coli*-infected calves at 14 days post-challenge. In humans, *N. massiliensis* is associated with intestinal dysbiosis and the pathogenesis of inflammatory bowel disease [29]. Further research is needed to better understand the specific role of *N. massiliensis* in growth performance of broilers.

*Acutalibacter* is a genus of the *Oscillospiraceae* family that also shows a negative correlation with BW of broilers of both sexes in this study. Currently, little is known about *Acutalibacter. N. timonensis*, a closely related species, was isolated earlier from the fecal sample of a human patient with type 2 diabetes (DSM 102082) [30], but its involvement in any host physiological functions is yet to be reported.

In contrast to those bacteria that are negatively associated with BW, *Anaerobutyricum,* a genus of the *Lachnospiraceae* family, is enriched in high-BW birds. One strain is positively associated with BW in both sexes, while another *Anaerobutyricum* strain appears to exert a beneficial role only in females. *Anaerobutyricum,* such as *A. hallii* and *A. soehngenii,* produces butyrate, but unlike other well-known butyrate-producing bacteria that convert complex oligo- and polysaccharides to butyrate, *Anaerobutyricum* spp. are considered lactate-utilizers, which rely on cross-feeding interactions to obtain lactate as their substrate [31]. These bacteria have specialized lactate utilization gene clusters, which allow them to overcome the energetic barrier of utilizing D, L-lactate to produce butyrate and propionate [32,33]. The D- and L-forms of lactate are important fermentation metabolites produced by intestinal bacteria, but are found to negatively affect mucosal barrier function and human health [34]. In addition, *A. hallii* is capable of producing pseudovitamin B12 [35], which is known as a modulator in shaping the structure and function of the human intestinal microbial community [36]. The enrichment of *Anaerobutyricum* in high-BW chickens may imply its potential as a probiotic for growth promotion in broilers.

Additionally, we have revealed *Clostridium fessum* F54 to be enriched in the high-BW group and positively correlated with BW only in males. *Clostridium fessum* belongs to the *Lachnospiraceae* family and was originally isolated from a human stool sample [37]. A unique feature of this bacterium is its inability to utilize most of its carbon sources to produce SCFAs, except for D-glucose and L-arabinose [37]. This is perhaps not surprising, given the fact that fast-growing broilers are normally provided with nutrient-dense diets normally devoid of complex carbohydrates. In fact, birds and broilers in particular maintain higher concentrations of glucose in the circulation than other vertebrates of similar BW including humans [38]. It is conceivable that any bacteria with the ability to directly convert glucose to SCFAs may be beneficial to gut health and growth performance. However, the beneficial impact of *Clostridium fessum* on animal growth and gut health needs to be experimentally verified.

The identification of BW-associated bacterial taxa represents a crucial initial step towards the development of probiotic formulations for BW management. The bacteria that are positively associated with BW could be incorporated into the diet or drinking water to potentially enhance animal growth. Conversely, the bacterial taxa that show a negative association with BW could be targeted for elimination through techniques such as genome editing [39] to minimize their undesirable influence on growth. However, such BW-reducing bacteria may hold promise for weight management interventions in humans. Consumption of these BW-reducing bacteria may have potential for the prevention and treatment of obesity, although additional research in humans and livestock animals is warranted to realize the potential.

## 5. Conclusions

We have confirmed structural differences in the intestinal microbiota among broilers of different BW. In addition, we have identified bacteria that are differentially enriched in high and low-BW broilers showing a positive or negative correlation with BW. Such associations may or may not be sex-dependent. The identification of the BW-associated bacteria provides important leads in developing potential probiotics to improve growth efficiency of chickens. Additionally, they may be explored as potential biomarkers for selection of chickens of different growth rates.

## Figures and Tables

**Table 1:** Pairwise comparisons of β-diversity of the cecal microbiota among different groups of day-42 Ross-708 broiler chickens.

Group	HM ^1^	MM	LM	HF	MF	LF
HM		0.053 ^2^ (0.100)	0.001 (0.234)	0.091 (0.256)	0.001 (0.242)	0.001 (0.289)
MM	0.005 (0.205)		0.013 (0.116)	0.010 (0.136)	0.062 (0.104)	0.009 (0.134)
LM	0.001 (0.206)	0.060 (0.048)		0.017 (0.082)	0.002 (0.117)	0.001 (0.122)
HF	0.001 (0.232)	0.511 (0.047)	0.113 (0.085)		0.002 (0.116)	0.001 (0.167)
MF	0.001 (0.294)	0.110 (0.102)	0.001 (0.218)	0.131 (0.083)		0.062 (0.094)
LF	0.001 (0.374)	0.498 (0.048)	0.028 (0.154)	0.071 (0.100)	0.001 (0.217)	

^1^ HM = high male, MM = medium male, LM = low male, HF = high female, MF = medium female, and LF = low female. ^2^ *p*-values and R^2^ (in parentheses) of pairwise comparisons of weighted (lower left panel) and unweighted UniFrac (upper right panel) distances of the cecal microbiota in different BW groups of male and female broilers were determined by PERMANOVA using 999 permutations.

## Data Availability

Raw sequencing reads of this study was deposited in the NCBI GenBank SRA database under the accession number PRJNA733787.
